# Hepatitis E Virus Infection after Platelet Transfusion in an Immunocompetent Trauma Patient

**DOI:** 10.3201/eid2301.160923

**Published:** 2017-01

**Authors:** Emmanuelle Loyrion, Thibaut Trouve-Buisson, Patricia Pouzol, Sylvie Larrat, Thomas Decaens, Jean-Francois Payen

**Affiliations:** Centre Hospitalier Universitaire Grenoble Alpes, Grenoble, France (E. Loyrion, T. Trouve-Buisson, P. Pouzol, S. Larrat, T. Decaens, J.-F. Payen);; Institut Albert Bonniot, Grenoble (T. Decaens);; Universités Grenoble Alpes, Grenoble (T. Decaens, J.-P. Payen);; Grenoble Institut des Neurosciences (GIN), Grenoble (J.-F. Payen)

**Keywords:** hepatitis E virus, platelet transfusion, trauma, injury, HEV, opportunistic infection, contaminated blood products, cholestasis, jaundice, blood transfusion, transfusion, immunocompetent, viruses

## Abstract

Hepatitis E virus (HEV) infection causes acute liver disease, but severe infections are rare in immunocompetent patients. We describe a case of HEV infection in a previously healthy male trauma patient in France who received massive transfusions. Genotyping confirmed HEV in a transfused platelet pool and the donor.

In developed countries, hepatitis E virus (HEV) infection usually results from consumption of contaminated meat or water and causes acute liver disease. Hepatitis E illness is usually self-limiting, and severe, prolonged infections are unusual except in immunocompromised patients. We describe HEV infection in a previously healthy man in France who received massive transfusions of blood, plasma, and platelets after a traumatic skiing accident. 

At hospital admission, the patient was in hemorrhagic shock caused by severe blunt splenic injury. He underwent an immediate splenectomy with massive transfusion: 9 packed red blood cells units, 7 fresh frozen plasma units, and 1 whole blood platelet pool. Because of hemopneumothorax, multiple rib fractures, and pulmonary contusions, severe acute respiratory distress syndrome developed, and the patient was treated with venovenous extracorporeal membrane oxygenation for 3 days. The patient received another whole blood platelet pool transfusion at day 5 posttrauma and was treated with renal replacement therapy for 6 weeks.

At day 15 posttrauma, the patient had icterus, and liver blood tests revealed cholestasis; ultrasound findings showed acalculous cholecystitis. Because bile drainage via percutaneous cholecystectomy was insufficient and led to septic shock (day 18), the patient underwent open cholecystectomy. The diagnosis of ulcerated cholecystitis was confirmed by histologic examination; *Enterococcus faecium* was isolated from blood and bile samples. Cholestasis, icterus, and cytolysis gradually resolved over the next week postoperatively; however, liver blood test results did not return to normal (Figure). Histological examination of the liver tissue from the biopsy performed during cholecystectomy gave normal results, and there was no evidence of drug-induced toxicity.

**Figure:**
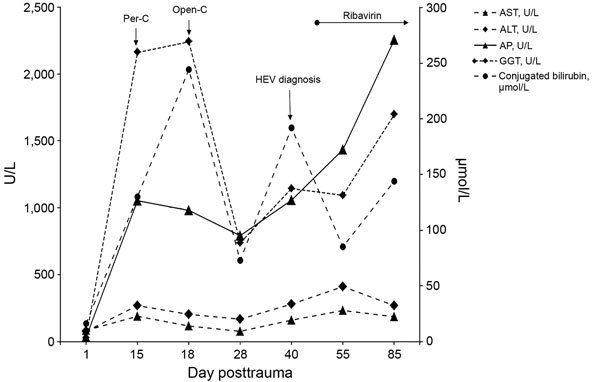
Time course of liver blood test results from a trauma patient in France who was transfused with an HEV-contaminated blood platelet pool on day 5 posttrauma. ALT, alanine aminotransferase; AP, alkaline phosphatase; AST, aspartate aminotransferase; GGT, gamma glutamyl transferase; HEV, hepatitis E virus; open-C, open cholecystectomy; per-C, percutaneous cholecystectomy.

At day 40, liver blood tests indicated a renewed increase in cholestasis and cytolysis (Figure). Meanwhile, the patient’s clinical condition had improved, renal function had recovered, and the tracheal tube was removed. Ultrasound examination of the abdomen showed normal findings. Viral serologic tests were negative for Epstein-Barr virus, herpes simplex virus, HIV, and hepatitis A, B, and C viruses. However, reverse transcription PCR testing revealed HEV positivity, with HEV viremia reaching 1.8 × 10^5^ copies/mL (Technical Appendix Table). Serologic tests for HEV IgM (ASSURE HEV IgM Rapid Test; MP Biomedicals, Singapore) and IgG (HEV IgG ELISA; Wantai, Coutaboeuf, France) were negative at that time, but a blood sample taken on day 75 posttrauma showed HEV IgM. 

The patient was not considered to be immunocompromised. Serologic tests for HIV were negative, leukocyte counts were within reference ranges, and no steroids were given to treat trauma. Neither the patient nor his family reported recent travel to HEV endemic areas or intake of uncooked or poorly cooked pork or game meat in the 3 months before the accident. All transfused blood products were retrospectively tested for HEV, and the blood platelet pool transfused at day 5 was identified as coming from an HEV-infected donor, who had viremia reaching 290 copies/mL. Evidence of direct blood contamination was provided by genotyping, which showed the virus in the donor and blood platelet pool were identical. HEV from the patient and the contaminated platelet pool were both HEV subtype 3f, and a phylogenetic study of the open reading frame 2 coding region by neighbor-joining cluster analysis confirmed the homology.

Because the patient was at high risk for severe acute HEV infection, treatment with 800 mg/day of ribavirin was initiated on day 45; the patient experienced severe nausea and vomiting but had no anemia. The course of the HEV infection reflected a slow response to the treatment. At 80 days posttrauma, the patient was still icteric with unchanged liver blood test results; HEV viremia was higher than before (2.34 × 10^7^ copies/mL; Technical Appendix Table). After 2 months on ribavirin treatment (day 110), HEV viremia started to decrease, but 3 months of treatment were needed for viremia levels to reach <100 copies/mL (day 135). 

The potential for HEV transmission by contaminated blood product transfusion, attributable to the high prevalence of HEV infection in asymptomatic blood donors, has been reported (*1*). Prevalence of HEV infection is ≈1/1,500 blood donations in Europe, and an HEV transmission rate as high as 42% was observed in immunocompromised patients given HEV-positive blood products (*2*). Chronic liver infections have developed in immunosuppressed patients (e.g., solid organ transplant recipients, patients with HIV infection, and patients with hematological disease) given HEV genotype 3–contaminated products (*3*,*4*). A few cases of prolonged HEV viremia in immunocompetent patients have been described (*5*,*6*), but none were transfusion-induced HEV infections in patients after trauma.

Successful treatments with ribavirin have been reported for transplant patients and patients with leukemia or HIV, including those at high risk for severe HEV infection (*7*). Treatment with ribavirin is usually characterized by rapid viral clearance (*8*) and ribavirin-induced anemia. Ribavirin could act by direct inhibition of viral replication or an immunomodulatory effect (*9*).

This case describes HEV infection acquired by an immunocompetent patient through transfusion of a contaminated blood product. Clinicians should consider the risk for HEV infection in trauma patients who receive large transfusions.

Technical Appendix Time course of hepatitis E virus viremia, IgM, and IgG levels in a trauma patient in France who was transfused with a contaminated blood platelet pool.
