# Supplementary material for: Hepatitis E Virus Infection after Platelet Transfusion in an Immunocompetent Trauma Patient
Source: Emerg Infect Dis. 2017 Jan;23(1):146–7. doi: 10.3201/eid2301.160923 (PMC5176217; doi:10.3201/eid2301.160923)
Supplement: Technical Appendix — Time course of hepatitis E virus viremia, IgM, and IgG levels in a trauma patient in France who was transfused with a contaminated blood platelet pool. [file 16-0923-Techapp-s1.pdf]

Article DOI: http://dx.doi.org/10.3201/eid2301.160923

Hepatitis E Virus Infection after Platelet Transfusion in an Immunocompetent Trauma Patient

Technical Appendix

Technical Appendix Table. Time course of HEV viremia, IgM, and IgG levels in a trauma patient who was transfused with an HEV-contaminated blood platelet pool on day 5 posttrauma*

Day posttrauma	Viral load (copies/mL)	HEV IgM	HEV IgG
40	1.8×10^5	-	-
75	2.34×10^7	+	-
110	486		
135	<100		

*HEV, hepatitis E virus.
